# The lesser trochanter profile is an accurate and reliable measure of femoral rotation for intramedullary nailing

**DOI:** 10.1051/sicotj/2024036

**Published:** 2024-09-20

**Authors:** Jack Mao, Malik Al-Jamal, David Allen, Brandon W. Henry, Tannor Court, Rahul Vaidya

**Affiliations:** 1 Wayne State University School of Medicine Detroit MI USA; 2 Michigan State University College of Osteopathic Medicine East Lansing MI USA; 3 Department of Orthopaedic Surgery, Detroit Medical Center Detroit MI USA

**Keywords:** Lesser trochanter profile, Femoral version, Malrotation, Intramedullary nailing

## Abstract

*Introduction*: The lesser trochanter profile (LTP) method is an intraoperative fluoroscopic technique that can assess the femoral version and limit malrotation. The purpose of this study was to directly assess the accuracy and reliability of the LTP method, as well as determine the incidence of malrotation produced by this technique. *Methods*: Three groups of observers (fellowship-trained orthopedic surgeons, orthopedic residents, and medical students) utilized the LTP method to replicate pre-imaged rotation angles on a cadaveric femur bone. Recorded outcomes include rotational error and number of attempts. Accuracy and interobserver reliability were assessed by rotational error and the interclass correlation coefficient (ICC), respectively. *Results*: Accuracy was within 3° for all three groups. ICC between each group was greater than 0.99. There was no statistical difference between the accuracy of fellowship-trained surgeons, orthopedic residents, and medical students. Medical students on average required more attempts to obtain their final image compared to fellowship-trained surgeons. There was no statistical difference in the number of attempts between residents and fellowship-trained surgeons. *Conclusion*: None of the LTP measurements were greater than 15°, the clinical threshold for malrotation. The average error of the observers was less than 3°, demonstrating that the LTP is an effective method of assessing the femoral version. There was no statistically significant difference between the observers, indicating that this technique is reliable and easy to use. Ultimately, the LTP method is easily reproducible for surgeons to avoid femoral malrotation.

## Introduction

Intramedullary nailing is a common treatment method for the fixation of diaphyseal femur fractures [1–4]. In a setting where cortical apposition is difficult to visualize on intraoperative fluoroscopy, malrotation can be a major concern [4–8]. Clinically, the femoral version ranges from −0.8° to 17.6° [9, 10]. In the operative setting, clinical malrotation has previously been defined as greater than 15° compared to the rotational state of the contralateral side [11]. Despite surgical advances, 20–40% of cases treated with intramedullary nailing still result in clinical malrotation with an anteversion difference greater than 15° [7, 12, 13]. This is a problem because malrotation of the femur can lead to abnormal rotational alignment of the entire extremity [13]. Importantly, this results in overall reduced hip function and can necessitate additional revision interventions [14–16].

Various fluoroscopic techniques have been utilized to measure femoral rotation and decrease the incidence of misalignment in the treatment of femoral shaft fractures [13, 17–20]. The most accurate method is to use intraoperative or postoperative CT scans with axial cuts of the femoral neck and condyles to measure the femoral version [8]. Another method, described by Tornetta et al., uses the true lateral of the femoral neck to measure the anteversion of the uninjured femur and replicate it on the contralateral side [21]. The Espinosa technique demonstrates that the inherent anteversion of the IM nail can also be used to set femoral rotation [22]. Yet another method, described by Kenawey et al., involves measuring the angle between the femoral head and greater trochanter to determine rotation [18]. The cortical step sign, described by Langer et al., compares and matches the cortical thickness and diameter of the bilateral femurs to prevent malrotation [13]. Finally, the lesser trochanter profile (LTP) method evaluates femoral rotation by using the lesser trochanter as a rotational landmark [20].

First described by Deshmukh et al., the LTP method is based on the idea that the protrusion of the LT correlates with the rotation angle of the femoral shaft [18]. On Anterior-Posterior (AP) X-ray imaging, the LT moves posteriorly to the femur as it internally rotates [14, 23]. This results in obstruction and the LTP appears to progressively decrease in size with more rotation [23]. Conversely, as the femur externally rotates, the LTP appears larger as the bony prominence becomes more perpendicular to the radiographic imaging [18]. In the described technique, the correct femoral version is achieved by matching the shape of the LT on the injured femur to that of the native, contralateral LTP [19, 20].

The current literature lacks sufficient research directly characterizing the reliability and accuracy of the LTP technique. There are few studies on the incidence of malrotation of more than 15° using this method. Additionally, no studies examined the level of experience required for this technique. These statistics are important because they measure the effectiveness of the LTP technique in preventing clinical malrotation during intramedullary nailing. This study measures the accuracy and reliability of the technique and evaluates the utility of the LTP method as a tool to avoid clinical malrotation.

## Methods and materials

### Study design

Three groups of observers (fellowship-trained orthopedic trauma surgeons, orthopedic surgery residents, and medical students) were recruited to use the LTP method to replicate the femoral version at five different rotation angles. For each rotation, the observer was allowed as many rotations as they wished until they were satisfied that they had matched the reference rotation. Subsequently, their number of attempts as well as the final rotation angle was recorded. The participants performed measurements independently of each other and were blinded to their results as well as those of their fellow observers.

### Experimental setup

**Figure 1:**
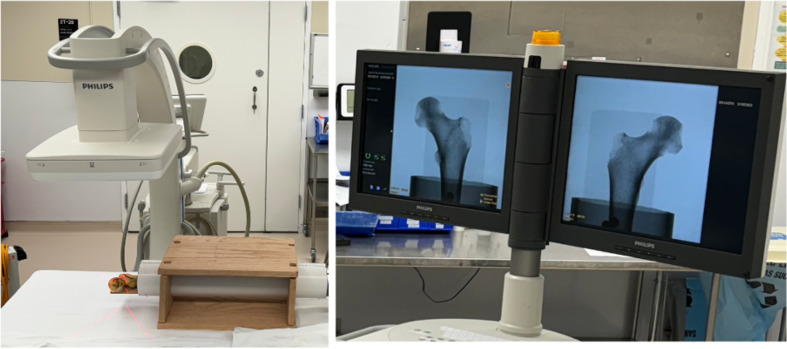
*Experimental Setup*: The cadaveric bone specimen was positioned on a radiographic table in a manufactured rotation device made of a radiopaque plastic pipe and a box. A C-arm fluoroscopy machine (Phillips Zendition 70, Eindhoven, NL) was centered 12 inches above the femoral head for imaging of the Lesser Trochanter (LT) profile. Observers rotated the pipe at the distal end to adjust rotation angle of the bone until they were satisfied that they had matched the reference image.

Measurements for this study were conducted on a left cadaveric femur bone. Radiographic imaging was obtained using C-arm fluoroscopy (Zendition 70, Phillips, Andover, MA, USA) on a radiographic table. The femur was placed within a manufactured bone rotation device (Figure 1), which was designed to allow for bone fixation and imaging at any designated angle.

**Figure 2:**
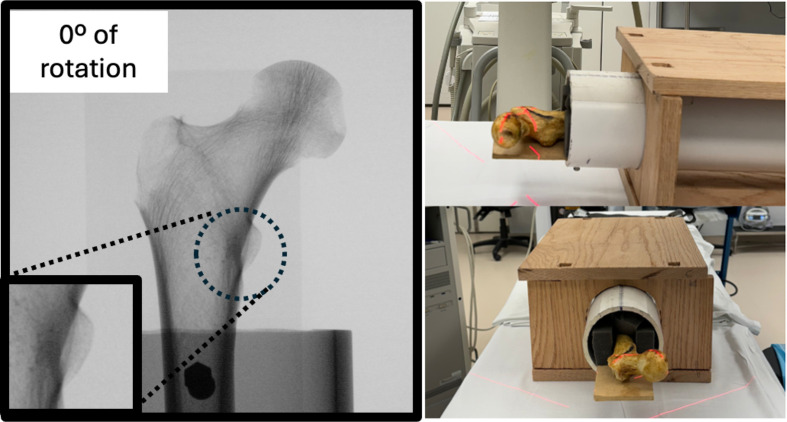
*Imaging Setup of the Lesser Trochanter in Neutral Position*: Observers obtained images with the femoral head as the fluoroscopic center using the C-arms laser aiming arm. The setup for imaging of the femoral head at 0° of rotation is shown.

**Figure 3:**
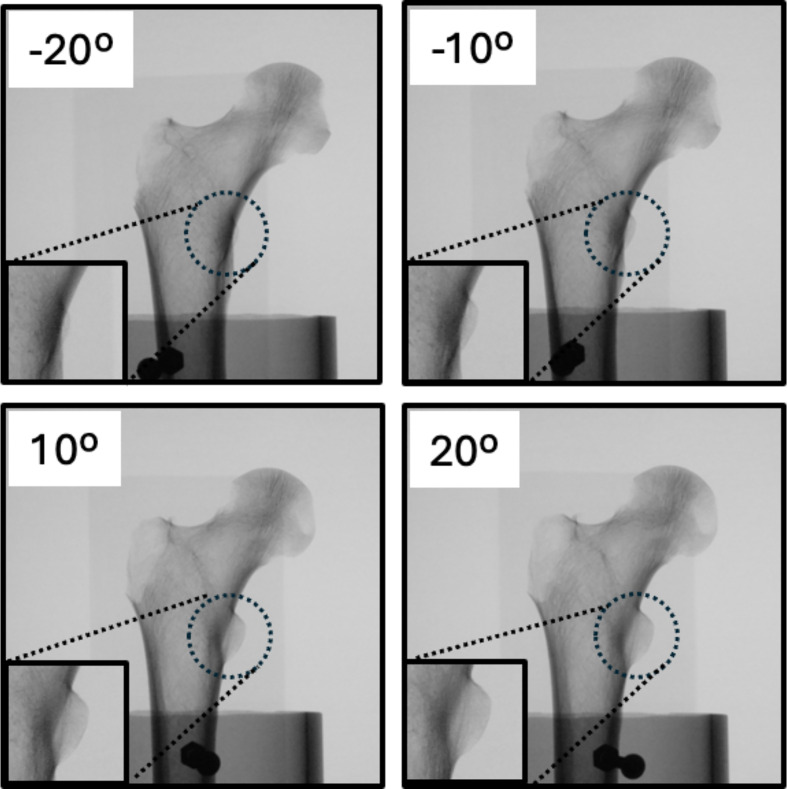
*Lesser Trochanter Profile View of Proximal Femur*: In addition to the 0° native rotation, the femur was imaged at four rotational angles to serve as reference for replication using the LTP method. The lesser trochanters are magnified in this figure for better visualization.

Following placement in the rotation device, the proximal femur was measured at various angles of version (−20° to +20°). The C-arm was positioned 12 inches from the bone, and the femoral head was set as the fluoroscopic center using the C-arm’s laser aiming arm (Figure 2). Fluoroscopy images were taken at 10-degree increments of the femoral version (−20°, −10°, 0°, +10, +20°) using an electronic protractor application (iPhone XR, Apple Inc., Cupertino, CA, USA) to ensure degree accuracy (Figure 3). The device was placed at the distal end of the femur rotation instrument and the femur was then normalized to neutral rotation before each attempt. Neutral rotation was defined to be when the posterior aspect of both femoral condyles was in contact with the stable base within the PVC pipe. Using the C-arm’s built-in software program, images were inverted left-to-right to be used as a reference.

### Statistical analysis

An absolute error was calculated by recording the difference between the measured final angle and the reference angle. A single-factor ANOVA test was performed to evaluate significant differences in the absolute mean rotation error as well as the number of attempts between observation groups. The intraclass correlation coefficient (ICC) and 95% confidence intervals (CIs) were calculated between observers in each group using a two-way mixed model with a significance level of 0.05.

## Results

The interobserver reliability of the LTP method was assessed using the ICC. ICC values were calculated to be 0.998 (95% CI, 0.990–1.000) for medical students, 0.994 (95% CI, 0.972–0.999) for residents, and 0.990 (95% CI, 0.950–0.999) for fellowship-trained surgeons (Table 1). ICC values above the level of 0.9 are considered excellent reliability, this was achieved by all observers within this study.

**Table 1:** Average intraclass correlation coefficient of each group in reproducing each of the reference angles.

Medical students	Orthopedic residents	Orthopedic surgeons
ICC (95% CI)	ICC (95% CI)	ICC (95% CI)
0.998 (0.990–1.000)	0.994 (0.972–0.999)	0.990 (0.950–0.999)

The accuracy of the LTP method was evaluated using the error between measured and reference values. Across the medical students, orthopedic residents, and fellowship-trained surgeons, replication of femoral rotation using the LTP technique yielded an absolute mean error of 1.0° ± 0.2°, 1.3° ± 0.8°, and 1.7° ± 0.9°, respectively (Table 2). There was no statistically significant difference in the accuracy between the fellowship-trained surgeons, residents, and medical students (*p* < 0.05).

**Table 2:** Average error of each group in reproducing each of the reference angles.

Reference angle	Medical students	Orthopedic residents	Orthopedic surgeons
	Mean error **±** SD	Mean error **±** SD	Mean error **±** SD
−20°	1.2° **±** 0.4°	0.4° ± 0.4°	2.2° ± 3.6°
−10°	0.5° ± 0.5°	0.6° ± 0.2°	1.0° ± 0.9°
0°	0.7° ± 0.7°	1.3° ± 0.8°	2.8° ± 1.0°
10°	1.1° ± 0.6°	2.0° ± 1.4°	1.4° ± 1.2°
20°	1.5° ± 0.3°	2.3° ± 2.8°	1.3° ± 0.4°
Overall	1.0° ± 0.2°	1.3° ± 0.8°	1.7° ± 0.9°

Femoral malrotation is defined as a difference of > 15° from the native femoral state [20]. Across all groups, there were no measurements that met this criterion for malrotation. Accuracy was within 3° for fellowship-trained surgeons, orthopedic residents, and medical students.

To assess reproducibility and ease of use, the number of attempts to arrive at the final image was also recorded for all observers. Across the medical students, orthopedic residents, and fellowship-trained surgeons, the mean number of attempts was 9.9 ± 4.0, 7.7 ± 2.9, and 4.9 ± 0.7, respectively (Table 3). Medical students on average required more attempts to obtain their final image compared to fellowship-trained surgeons (*p* < 0.05). There was no statistical difference in the number of attempts between the medical students and residents (*p* < 0.05) or between residents and fellowship-trained surgeons (*p* < 0.05).

**Table 3:** Average number of attempts of each group in reproducing each of the reference angles.

Reference angle	Medical students	Orthopedic residents	Orthopedic surgeons
	# attempts ± SD	# attempts ± SD	# attempts ± SD
−20°	12.3 ± 7.5	9.0 ± 2.6	5.0 ± 0.0
−10°	9.7 ± 5.5	9.3 ± 4.2	5.7 ± 2.1
0°	10.3 ± 3.0	6.7 ± 5.5	5.3 ± 0.6
10°	8.0 ± 4.6	8.0 ± 3.6	3.3 ± 0.6
20°	9.3 ± 9.3	5.7 ± 0.6	5.0 ± 1.0
Overall	9.9 ± 4.0	7.7 ± 2.9	4.9 ± 0.7

## Discussion

Malrotation of the femur greater than 15° is associated with clinical consequences such as functional impairment of daily activities and degenerative arthritis [14, 16]. The incidence of malrotation after intramedullary nailing of femur fractures is estimated to be between 20–30% [7]. Various methods have been described to assess femoral rotation [24, 25]. The gold standard is to use CT scans intraoperatively or postoperatively to evaluate malrotation [8]. The Espinosa technique is widely used to set femoral rotation with the inherent anteversion of the IM nail [22], and Tornetta’s technique is very reliable as well in determining the version using the true lateral and femoral necks [21]. This study analyzed the LTP method as an alternative method of obtaining femoral rotation.

The LTP method is a way of evaluating the femoral version to prevent clinical malrotation after intramedullary nailing [23], but there is a lack of research that directly assesses the accuracy and reliability of this technique. In this study, we directly evaluate the LTP technique through fluoroscopic imaging. The findings show that the LTP is reliable with an excellent ICC score. Additionally, it is accurate to within 3°. Finally, experience varies directly with the number of attempts.

The results of this study show that the LTP technique is reliable and accurate, making it an effective tool to prevent malrotation. Each of the three groups of observers was able to make measurements that exceeded an ICC of 0.99, nearly at a perfect ICC coefficient of 1. Thus, these findings demonstrate that the LTP technique is a reliable method to replicate the femoral rotation, despite our initial hypothesis. Moreover, using the LTP method to replicate femoral version resulted in a mean rotational error of less than 3°, the highest being only 1.7° ± 0.9°. This indicates that the technique is an accurate method of evaluating femoral rotation. Of note, the average number of attempts for fellowship-trained surgeons, orthopedic residents, and students was 4.9, 7.7, and 9.9, respectively. The medical students used more attempts than fellowship-trained surgeons but there was no statistical difference between residents and surgeons. This likely reflects the efficiency of the more experienced groups of observers.

One limitation of this study is that it only tested measurements of the proximal hip. To obtain the absolute anteversion would involve imaging the distal femurs on the lateral view [8]. However, this did not matter, since the LTP method involves comparing rather than measuring absolute torsional angles [20]. Moreover, we believe that most of the variation in femoral rotation originates from the proximal hip, which is why the LTP technique is such an accurate tool. Another limitation of this study is the in-vitro design. All experiments were conducted on a cadaveric femur bone without soft tissue attachments. In the context of intraoperative fluoroscopic imaging with a real live patient, the visualization of the proximal hip could potentially be different.

The femoral version is important because malrotation greater than 15° leads to clinical consequences such as hip and knee pain [19]. While there are various modalities of measuring rotation such as ultrasound and CT, many of these methods have drawbacks [7]. The Tornetta technique is accurate however it adds about 10–15 min to intraoperative time [21]. The LTP method addresses these challenges because it is a fluoroscopic technique that can be employed intraoperatively without prolonging the operation time. Moreover, because the technique relies on a comparative approach to measuring version, it is easier to use and does not require extra instruments [20]. As this study showed, the LTP technique is accurate and reliable, and its efficacy does not depend on operator experience, making it a good option to measure the femoral version and avoid malrotation.

## Conclusion

The LTP method is an effective technique for recreating proximal femoral rotation, irrespective of the practitioner’s experience level. The method’s consistent performance, evidenced by low average errors and high ICC values across all groups, underscores its applicability in clinical settings.

## Data Availability

The data that supports the findings of this study are available upon reasonable request.

## References

[1] Winquist RA, Hansen ST Jr, Clawson DK (1984) Closed intramedullary nailing of femoral fractures. A report of five hundred and twenty cases. J Bone Joint Surg Am 66(4), 529–539. 6707031

[2] Hudson I, Mauch K, Schuurman M, Padela MT, Gheraibeh P, Vaidya R (2019) Effect of inherent tibial asymmetry on leg length discrepancy measurements after intramedullary nailing of comminuted femoral shaft fractures. SICOT J 5, 1–6. 30632481 10.1051/sicotj/2018053 PMC6329309

[3] Ulici A, Odagiu E, Haram O, Ionescu A, Sterian GA, Carp M, Tevanov I (2020) Poor prognostic factors of femoral shaft fractures in children treated by elastic intramedullary nailing. SICOT J 6, 34. 32870156 10.1051/sicotj/2020031 PMC7461699

[4] Bråten M, Terjesen T, Rossvoll I (1995) Femoral shaft fractures treated by intramedullary nailing. A follow-up study focusing on problems related to the method. Injury 26(6), 379–383. 7558257 10.1016/0020-1383(95)00054-d

[5] Kent ME, Arora A, Owen PJ, Khanduja V (2010) Assessment and correction of femoral malrotation following intramedullary nailing of the femur. Acta Orthop Belg 76(5), 580–584. 21138210

[6] Gugenheim JJ, Probe RA, Brinker MR (2004) The effects of femoral shaft malrotation on lower extremity anatomy. J Orthop Trauma 18(10), 658–664. 15507818 10.1097/00005131-200411000-00002

[7] Jaarsma RL, van Kampen A (2004) Rotational malalignment after fractures of the femur. J Bone Joint Surg Br 86(8), 1100–1104. 15568519 10.1302/0301-620x.86b8.15663

[8] Branca Vergano L, Coviello G, Monesi M (2020) Rotational malalignment in femoral nailing: prevention, diagnosis and surgical correction. Acta Biomed 91, 1–11. 10.23750/abm.v91i14-S.10725 PMC7944689 33559631

[9] Litrenta JM, Domb BG (2018) Normative data on femoral version. J Hip Preserv Surg 5(4), 410–424. 30647933 10.1093/jhps/hny048 PMC6328757

[10] Hetsroni I, Dela Torre K, Duke G, Lyman S, Kelly BT (2013) Sex differences of hip morphology in young adults with hip pain and labral tears. Arthroscopy 29(1), 54–63. 23200844 10.1016/j.arthro.2012.07.008

[11] Bråten M, Terjesen T, Rossvoll I (1993) Torsional deformity after intramedullary nailing of femoral shaft fractures. Measurement of anteversion angles in 110 patients. J Bone Joint Surg Br 75(5), 799–803. 8376444 10.1302/0301-620X.75B5.8376444

[12] Citak M, Suero EM, O’Loughlin PF, Arvani M, Hüfner T, Krettek C, Citak M (2011) Femoral malrotation following intramedullary nailing in bilateral femoral shaft fractures. Arch Orthop Trauma Surg 131(6), 823–827. 21191605 10.1007/s00402-010-1245-6

[13] Langer JS, Gardner MJ, Ricci WM (2010) The cortical step sign as a tool for assessing and correcting rotational deformity in femoral shaft fractures. J Orthop Trauma 24(2), 82–88. 20101131 10.1097/BOT.0b013e3181b66f96

[14] Marchand LS, Todd DC, Kellam P, Adeyemi TF, Rothberg DL, Maak TG (2018) Is the lesser trochanter profile a reliable means of restoring anatomic rotation after femur fracture fixation? Clin Orthop Relat Res 476(6), 1253–1261. 29470236 10.1007/s11999.0000000000000226 PMC6263571

[15] Naqvi G, Stohr K, Rehm A (2017) Proximal femoral derotation osteotomy for idiopathic excessive femoral anteversion and intoeing gait. SICOT J 3, 49. 28675371 10.1051/sicotj/2017033 PMC5496450

[16] Abubeih HMA, Farouk O, Abdelnasser MK, Eisa AA, Said GZ, El-Adly W (2018) Femoral malalignment after gamma nail insertion in the lateral decubitus position. SICOT J 4, 34. 30058530 10.1051/sicotj/2018033 PMC6065270

[17] Espinoza C, Sathy AK, Moore DS, Starr AJ, Reinert CM (2014) Use of inherent anteversion of an intramedullary nail to avoid malrotation in femur fractures. J Orthop Trauma 28(2), 34–38. 10.1097/BOT.0b013e318298e48c 23689227

[18] Kenawey M, Krettek C, Ettinger M, Hankemeier S, Breitmeier D, Liodakis E (2011) The greater trochanter-head contact method: a cadaveric study with a new technique for the intraoperative control of rotation of femoral fractures. J Orthop Trauma 25(9), 549–555. 21654528 10.1097/BOT.0b013e3181f9eeac

[19] Jaarsma RL, Ongkiehong BF, Grüneberg C, Verdonschot N, Duysens J, van Kampen A (2004) Compensation for rotational malalignment after intramedullary nailing for femoral shaft fractures. An analysis by plantar pressure measurements during gait. Injury 35(12), 1270–1278. 15561117 10.1016/j.injury.2004.01.016

[20] Deshmukh RG, Lou KK, Neo CB, Yew KS, Rozman I, George J (1998) A technique to obtain correct rotational alignment during closed locked intramedullary nailing of the femur. Injury 29(3), 207–210. 9709422 10.1016/s0020-1383(97)00182-4

[21] Tornetta P 3rd, Ritz G, Kantor A (1995) Femoral torsion after interlocked nailing of unstable femoral fractures. J Trauma 38(2), 213–219. 7869438 10.1097/00005373-199502000-00011

[22] Espinoza C, Sathy AK, Moore DS, Starr AJ, Reinert CM (2014) Use of inherent anteversion of an intramedullary nail to avoid malrotation in femur fractures. J Orthop Trauma 28(2), 34–38. 10.1097/BOT.0b013e318298e48c 23689227

[23] Jaarsma RL, Verdonschot N, van der Venne R, van Kampen A (2005) Avoiding rotational malalignment after fractures of the femur by using the profile of the lesser trochanter: an in vitro study. Arch Orthop Trauma Surg 125(3), 184–187. 15688229 10.1007/s00402-004-0790-2

[24] Ivanov DV, Welby JP, Khanna A, Barlow JD, Sems SA, Torchia ME, Yuan BJ (2024) Evaluation of intraoperative fluoroscopic techniques to estimate femoral rotation: a cadaveric study. J Orthop Trauma 38(5), 279–284. 38381135 10.1097/BOT.0000000000002790

[25] Jang ES, Davignon R, Geller JA, Cooper HJ, Shah RP (2024) Accuracy of the Lesser Trochanter Profile as a Marker of Femoral Rotation: Computed Tomography-Based Study of 1,722 Femora. J Bone Joint Surg Am 106(10), 912–918. 38381806 10.2106/JBJS.23.01052

